# For the Farm and Family

**Published:** 1879-09

**Authors:** 


### FOR THE FARM AND FAMILY.

The following recipes for metals resembling gold are said to produce a metal which will so nearly approximate the genuine as almost to defy detection, without a resort to thorough tests: Fuse, together with saltpetre, sal ammoniac, and powdered charcoal, 4 parts platinum, 2½ parts pure copper, 1 part pure zinc, 2 parts block tin, and 1½ parts pure lead. Another good recipe calls for 2 parts platinum, 1 part silver, and 3 parts copper.

Cement for sealing fruit cans is made of resin 1 lb., tallow 1 oz.

In using Paris green to exterminate the potato bugs, the poison should be mixed with the cheapest grade of flour, 1 lb. of green to 10 of flour. A good way of applying it to the plants is to take an old 2 quart tin fruit can, melt off the top, and put in a wooden head, in which insert a broom handle. Bore a hole in the head, also, to pour the powder in, and then punch the bottom full of holes about the size of No. 6 shot. Walk alongside the rows, when the vines are wet with dew or rain, and make one shoot at each hill.

In some parts of the country there have been large numbers of the orchard or tent caterpillars, which have left their rings of eggs on the young twigs. If these are now cut off with a clipping pole, it will prevent in every instance a large nest of caterpillars, and be much more easily done than after the latter have grown.

Equal proportions of turpentine, linseed oil, and vinegar, thoroughly applied, and then rubbed with flannel, is an excellent furniture polish.

The alloy popularly known as oroide, from which a large number of cheap watches, chains, and trinkets are now manufactured, is made of pure copper 100 parts, tin 17 parts, magnesia 16 parts, sal ammoniac ¼ part, quicklime ⅛ part, tartar of commerce 9 parts. The copper is first melted, then the magnesia, sal ammoniac, lime, and tartar in powder are added little by little, and briskly stirred for half an hour. The tin is lastly mixed in in grains until all is fused. The crucible is covered, and the fusion maintained for 35 minutes, when the dross is skimmed off, and the alloy is ready for use.

A simple mode of keeping butter in warm weather is to set over the dish containing it a large flower pot or unglazed earthenware crock, inverted. Wrap a wet cloth around the covering vessel, and place the whole where there is a draft of air.

Rats detest chloride of lime and coal tar.

White horn buttons may be made to imitate mother of pearl by being boiled in a saturated solution of sugar of lead, and then laid in very dilute hydrochloric acid.

The following is a simple way of obtaining copies of writing without the use of a copying press: Mix white sugar with the ink, 1½ drachms sugar to 1 oz. ink. Use this with an ordinary pen, and place over the writing a moistened sheet of unsized paper. Lay both leaves between two layers of carpet; put the whole under a piece of board large enough to cover. Then stand on the board for a few seconds. An excellent impression will be found on the copying paper.

To extract rust from steel, immerse the article to be cleaned in a solution of ½ oz. cyanide of potassium to a wine glass full of water, until the dirt and rust disappear. Then clean by means of a tooth brush with a paste composed of cyanide of potassium, castile soap, whitening. and water.

Awnings can be rendered waterproof by plunging the fabric into a solution containing 20 per cent. of soap, and afterwards into another solution containing the same percentage of sulphate of copper. Wash, and the operation is finished.

The best pine wood evaporates 5 lbs. of water per lb. wood, consumed in a steam boiler furnace. One cord of wood can be consumed per hour on 60 square feet of grate. One pound carbon burnt to carbonic acid requires the oxygen of 153 cubic feet of atmospheric air.

Iron filings in a weak solution of sal ammoniac, mixed with Portland cement, are said to double the strength of the latter.

The following compounds are useful for soldering or tinning: Tin, 1 part muriatic acid, with as much zinc as it will dissolve; add 2 parts water and some sal ammoniac. Brass and copper, 1 lb. muriatic acid, 4 ozs. zinc, 5 ozs.

